# Matrix Metalloproteinase in Abdominal Aortic Aneurysm and Aortic Dissection

**DOI:** 10.3390/ph12030118

**Published:** 2019-08-06

**Authors:** Eithne M. Maguire, Stuart W. A. Pearce, Rui Xiao, Aung Y. Oo, Qingzhong Xiao

**Affiliations:** 1 Centre for Clinical Pharmacology, William Harvey Research Institute, Barts and The London School of Medicine and Dentistry, Queen Mary University of London, London EC1M 6BQ, UK; 2 Barts Health NHS Trust and the Barts NIHR Biomedical Research Centre, William Harvey Research Institute, Barts and The London School of Medicine and Dentistry, Queen Mary University of London, London EC1M 6BQ, UK

**Keywords:** matrix metalloproteinase, abdominal aortic aneurysm, aortic dissection, aortic disease, inflammation

## Abstract

Abdominal Aortic Aneurysm (AAA) affects 4–5% of men over 65, and Aortic Dissection (AD) is a life-threatening aortic pathology associated with high morbidity and mortality. Initiators of AAA and AD include smoking and arterial hypertension, whilst key pathophysiological features of AAA and AD include chronic inflammation, hypoxia, and large modifications to the extra cellular matrix (ECM). As it stands, only surgical methods are available for preventing aortic rupture in patients, which often presents difficulties for recovery. No pharmacological treatment is available, as such researchers are attempting to understand the cellular and molecular pathophysiology of AAA and AD. Upregulation of matrix metalloproteinase (MMPs), particularly MMP-2 and MMP-9, has been identified as a key event occurring during aneurysmal growth. As such, several animal models of AAA and AD have been used to investigate the therapeutic potential of suppressing MMP-2 and MMP-9 activity as well as modulating the activity of other MMPs, and TIMPs involved in the pathology. Whilst several studies have offered promising results, targeted delivery of MMP inhibition still needs to be developed in order to avoid surgery in high risk patients.

## 1. Introduction

Abdominal aortic aneurysms (AAA) and aortic dissection (AD) are devastating conditions occurring within the aortic wall, which if left unchecked, lead to extensive dilatation of the major blood vessel and eventually aortic rupture. Aortic rupture can lead to adverse consequences, with low survival rates and elevated mortality rates for survivors of aortic rupture [1,2]. Importantly, the term ‘aneurysm’ refers to the abnormal enlargement of the aorta, whereas the term ‘aortic dissection’ refers to the process of intimal tearing within the aortic wall. This last process allows blood to pool within the region, creating recirculating blood flow and forming a ‘false lumen’ [3]. Aneurysm formation and aortic dissection occur as a result of drastic changes to the structure of the aortic wall. These changes occur due to steady degradation of the extracellular matrix (ECM) proteins, chronic inflammation and oxidative stress [4]. However, the underlying pathophysiological processes responsible for these two conditions have not yet been fully elucidated. As our understanding improves, new drugs targets may develop to tackle these pathophysiological events. Tissue sections and blood samples taken from AAA and AD patients have helped researchers to identify key member of the matrix metalloproteinase (MMP) family, which become upregulated in these diseases [5]. Studies in mice have helped us to gain a greater knowledge of how these MMPs are regulated and how they operate in situ to bring about extensive changes in ECM composition of the aorta. This Review will examine how research into the role of MMPs in AAA and AD may offer a new pharmacological route for reducing the rate of development of these pathologies, and avoid the calamitous consequences of aortic rupture.

## 2. Epidemiology of AAA and AD

An AAA is typically defined as an enlargement of the aortic wall with an aortic diameter equal to or greater than 3 cm [6]. It affects approximately 4–8% of the population [7]. Risk factors for AAA development include; smoking [8], advanced age (particularly in the over-70s male population) [9,10,11], a family history of AAA, and cardiovascular disease [12,13,14]. Various co-morbidities are associated with AAA, including myocardial infarction and peripheral vascular disease in both men and women, and stroke in women only [15]. Of note, there is a much higher risk of developing AAA in men compared to women [16], but a greater risk of AAA rupture in women [17]. Usually asymptomatic, an AAA can go undetected up until the point of rupture, which has a high fatality rate [18]. Thus, early screening programs for AAA have been adopted in the United Kingdom since 2009 for men aged 65 and over, and have since achieved a significant drop in the prevalence of AAA rupture [19].

Imaging techniques required to diagnose and monitor aneurysmal development in small AAAs include abdominal ultrasound and duplex ultrasonography, whereas computed tomography (CT) angiography is required to scan aneurysm above the renal arteries during follow up and before any treatment [20]. Magnetic resonance imaging is less routinely used, but offers and advantage over other imaging techniques such as no radiation exposure. Finally, positron emission tomography-computed tomography can also be used for monitoring aneurysmal growth, particularly in inflammatory or infective aneurysm and associated pathologies [21].

Aortic rupture is typically treated either with open repair or endovascular aneurysm repair (EVAR) [22]. EVAR is also used for elective repair in patients with infrarenal AAA with an aortic diameter greater than 5.5 cm [23] although the RESCAN study determined a diameter of 4.5 cm in women as a threshold for treatment, since the risk of rupture in women is four times higher than that in men [24]. Available techniques for elective repair also include open repair, requiring the administration of heparin to reduce the risk of thrombosis [25], which is the standard procedure for nonemergency infrarenal AAA according to the current 2019 guidelines [26], although such a procedure presents with significantly higher mortality risks compared with EVAR [27].

Unfortunately, EVAR is plagued by complications such as endoleaks, whereby inadequate sealing or placement of the stent graft leads to persistent blood flow outside of the graft, increasing the chance of aortic rupture. Migration of the stent graft >10 mm away from the assigned location can also result in endoleak, stent graft occlusion and stent graft separation [26]. In light of these complications, new techniques such as ‘endostapling’, which improves stent graft fixation, and multilayered stent grafts have been investigated for their improved treatment outcomes [14].

Pharmacological treatments such as doxyclicine and angiotensin-converting enzyme (ACE) inhibitors have been investigated for their potential to treat and/or prevent AAA growth, as well as reducing the likelihood of aortic rupture. However, both have been dismissed as they failed to adequately replicate findings from animal models, and showed conflicting outcomes in clinical studies respectively [28,29,30,31,32]. Currently, the European Society for Vascular Surgery recommends β-blockers, aspirin and statins for treating/preventing AAA growth or aortic rupture, despite no clear evidence for delayed aneurysmal growth [26].

As it stands, pharmacological means of treating AAA are sorely lacking, in part due to a poor understanding in how aneurysm formation and/or AD is initiated and progresses.

## 3. Cellular and Molecular Pathophysiology of AAA and AD

Although multiple processes occurring throughout the course of AAA and AD have been identified, the chronology of events has been difficult to establish in humans as most cases present mid to late stage in the disease. As such, various animal models of AAA and AD have been used to further our understanding including; the global angiotensin II-infusion model, the aortic periadventitial CaCl_2_ application model, and the intralluminal elastase infusion model [33]. Although each of these models replicate one or more key aspects of AAA pathology, they serve only to study the role of a specific cell type and its signaling pathways, and thus information gleaned with regards to early disease triggers are limited. Therefore, our current understanding of AAA progression is viewed within the context of multiple pathological events occurring simultaneously. Key events occurring within the aortic wall include inflammation, vascular smooth muscle cell (VSMC) apoptosis, oxidative stress, and ECM degradation.

It has long been established that inflammation plays a huge role in onset and progression of AAA. Elevated C-reactive proteins levels detected in AAA patients [34,35], as well as a large leukocyte presence in the aortic wall of AAA patients [36], provide a strong indication of immune system involvement in AAA, as well as AD [37] and rupture [38,39]. In 1972, Walker et al. described the ‘inflammatory aneurysm’ with ‘extensive active chronic inflammatory changes’ seen in tissue sections of aneurysm patients [40] with the inflammatory variant of AAA disease [41]. Of note, the more common variant known as ‘atherosclerotic’ AAA, is also marked by immune cell infiltration, however, without the excessive fibrosis or thickening of the aortic wall seen in 5–10% of aneurysm patients with ‘inflammatory’ AAA condition [41]. Importantly, cells of both the innate and adaptive arms of the immune system are observed within the adventitial and medial layers of the aortic wall during aneurysm development. Both neutrophils and macrophages have been identified as key players in AAA progression. Early neutrophil infiltration in aneurysm development was found to be essential in initiating immune cell recruitment through ECM remodeling, as neutrophil depletion studies showed a significant drop in AAA formation in mice with far smaller aortic diameters [42]. Their role in releasing neutrophil elastase [43] and forming neutrophil extracellular traps in response to elevated interleukin-1 β (IL-1β) levels [44], point to a vital function for neutrophils in the vast degradation of the ECM within the aortic wall [45]. Of note, neutrophil function in later stages of AAA progression has also been studied, with AD found to be mediated by neutrophil-derived MMP-9 release from neutrophils present in the intima of the aortic wall [46].

Equally macrophages in AAA, predominantly derived from circulating monocytes [47] have been shown to play a role in early AAA processes, particularly through the release of pro-inflammatory cytokines, such as tumor necrosis factor- α (TNF- α), and interferon- γ (IFN- γ) [48], which have been studied for their use as biomarkers for AAA [49]. Moreover, their recruitment to ECM injury sites and exposure to elastin breakdown products has been shown to trigger phenotype switching towards either the M1 ‘pro-inflammatory’ phenotype or the M2 ‘anti-inflammatory’ phenotype. M1 and M2 macrophages are predominantly localized in the adventitial layer and the intraluminal thrombus, respectively, highlighting separate roles for these macrophage subtypes in AAA [50]. Importantly, the M1/M2 ratio was found to be elevated in ruptured human cerebral aneurysms, compared to unruptured aneurysms where an equal ratio was observed [51].

Following innate immune cell infiltration, T cells, in particular CD4+ T helper cells play a huge role in aneurysm pathology. Indeed, depletion of CD4+ T cell populations prevents AAA development in mice, however, injection with the CD4+ T cell-derived cytokine, IFN- γ, can reconstitute AAA formation even in the absence of CD4+ T cells [52]. Importantly an imbalance in T helper subsets, Th1 and Th2, has been linked with AAA formation and development [53,54,55].

B cells have also been observed in the adventitial wall of aneurysmal tissue sections [56]. As an abundant source of immunoglobulins, complement and pro-inflammatory cytokines, immune cell recruitment is potentiated in part by B cells, which require activation through spleen tyrosine kinase (Syk). Furthermore, as an additional source of MMPs, inhibition of B cell activation using a Syk-specific inhibitor led to suppressed expansion of aneurysm tissue sections isolated from mice, with a concomitant reduction in inflammation and immunoglobulin activity [57].

Overall, various pro-inflammatory signaling pathways have been investigated as a potential way of preventing global inflammatory cell infiltration into the aortic wall. For instance, the c-Jun NH2-terminal protein kinase (JNK) pathway promotes AAA development by inducing pro-inflammatory chemokine release [58,59,60], such as monocyte chemoattractant protein-1 (MCP-1). It is typically activated through toll-like receptors (TLRs); TLR-2 and TLR-4, which are expressed on immune cells; macrophages and B cells, in AAA [61]. JNK pathway inhibition has been shown to reduce MMP production and chemokine-mediated macrophage migration, slowing the progression of AAA development in rats and humans [62,63,64]. Moreover, direct inhibition of TLR-2 and TLR-4 has also proven successful in reducing AAA formation and recurrence in mice and humans [65,66,67,68]. Finally, inhibition of another pro-inflammatory pathway, the nuclear factor kappa-light-chain-enhancer of activated B cells (NFkB) pathway in endothelial cells, has been shown to be beneficial in protecting mice from angiotensin II-induced AAA formation by reducing macrophage infiltration, oxidative stress and aortic inflammation [69]

In the presence of such large-scale inflammation, the aortic wall undergoes significant weakening, which is compounded by oxidative stress, VSMC apoptosis and ECM remodeling. With the upregulation of inducible nitric oxide synthase (iNOS) and the presence of nicotinamide adenine dinucleotide phosphate hydrogen (NADPH) oxidases, reactive oxygen species (ROS) are abundant in the aortic wall, resulting in further activation of ECM degrading enzymes and VSMC apoptosis. As expected, iNOS knock out (iNOS^-/-^) mice and mice treated with a selective NADPH oxidase inhibitor were found to be resistant to developing aneurysms, highlighting the crucial role that oxidative stress plays in aneurysm formation [70]. Importantly, the iNOS^-/-^ group expressed reduced levels of MMP-2 and MMP-9, two of the key MMPs involved in AAA and AD. Although ROS has also been shown to stimulate the secretion of cyclophilin A (CyPA) from VSMCs, which initiates VSMC migration and proliferation [71], elevated and sustained levels of ROS will eventually induce VSMC apoptosis [72], resulting in a depletion of cellular content of the medial layer of the aorta. Finally, the release of matrix degrading enzymes by immune cells present in the inflammatory milieu of the aortic wall, add to the extensive changes seen in ECM. Early elastin fragmentation followed by collagen degradation, are hallmarks of AAA and AD pathology, and considered crucial for aortic dilation and eventual rupture [73]. Protein degrading enzymes such as urkinase-type plasminogen activator (u-PA) [74] and a host of MMPs are released into the weakened wall, thereby perpetuating the pathology [75,76]. Halting the destruction of vital structural components of the ECM is key to salvaging the integrity of the aorta and preventing rupture. As such, developing pharmacological inhibitors of MMPs may prove useful in determining the course of AAA and AD progression.

## 4. MMP and TIMPs Overview

MMPs are a class of zinc-dependent endopeptidase proteins [77], with roles in both physiological processes, such as angiogenesis [78], and pathological diseases such as cancer [79] and cardiovascular disease [80]. The MMP family consists of 28 endopeptidases, which must be enzymatically activated before they can begin degrading various components of the ECM either through cleavage by other MMPs and proteinases or activation through their catalytic domain via thiol modifying agents and ROS [81].

Originally categorized based on the substrate they degrade, namely collagenases (MMP-1, MMP-8, MMP-13, MMP-18), stromolysins (MMP-3 and MMP-10), gelatinases (MMP-2 and MMP-9) and matrilysins (MMP-7 and MMP-26), the discovery of more MMP family members paved the way for a new numbering system, based on molecular structure [82]. Each MMP contains a conserved signal peptide along with a pro-domain and a catalytic domain, differing within each sub-group. Added regions are present and required for the breakdown of specific substrates and are often a determining factor in the localization and function of the MMP. For instance, the hinge region of MT-MMP1 (MMP-14) plays a key role in pericellular proteolysis by enabling autocatalytic processing of MT-MMP1 [83]. MMP substrates include ECM proteins (type I/IV collagen, gelatin, laminin, fibronectin), cell adhesion molecules (E-cadherin), inflammatory cytokines (monocyte chemoattractant protein 1/3) and breakdown products (IL-1β degradation) [84]. Importantly, MMP expression is regulated by endogenous tissue inhibitors of MMPs (TIMPs). Appropriate MMP activity hinges on adequate TIMP expression, and an irregular MMP/TIMP ratio may lead to excessive ECM breakdown, as is the case in AAA and AD pathology [85].

In particular, MMP-9 and MMP-1 levels were found to be significantly upregulated in aneurysmal aortic specimens compared to healthy aortic tissues. Moreover, the ratio of MMP to TIMP expression was found to be higher in diseased specimens [86]. Of greater interest is the relationship between aneurysm size and the abundance of specific MMPs at different stages of disease progression. Intriguingly, MMP-9 expression was found to be correlated with aneurysm diameter (over 5 cm), implying a role for this MMP in later stages of the pathology. Equally another gelatinase, MMP-2, has been implicated in AAA pathology, as elevated expressions of its activated form were found bound to the ECM of tissues isolated from AAA patients [87]. As such, both MMPs have been of considerable interest in the pathology of AAA and AD, and may offer pharmacological means of preventing or slowing these pathologies.

## 5. Clinical and GWAS Studies: MMPs in AAA and AD

As evidenced above, AAA and AD are complex pathologies whose outcome relies on cell-mediated processes regulating MMP activity and behavior. In addition to which, underlying genetic risk factors also play an important role in influencing AAA and AD likelihood and outcome. Increased susceptibility for AAA has been determined for several gene variants including in the DAB2IP gene, which encodes an inhibitor of cell growth and survival [88], and low-density lipoprotein receptor (LDLR) [89]. Both of these variants showed association with additional cardiovascular conditions such as peripheral artery disease and coronary artery disease, respectively. By contrast, the single nucleotide polymorphism (SNP) in the low-density-lipoprotein receptor-related protein 1 (LRP1), rs1466535, was found to be specifically associated with AAA only [90].

This finding points to a pathological deviation of AAA and AD from other CVD diseases, which share similar pathologies. Gene variants in sortilin-1 (SORT1) [91] and interleukin-6 receptor (IL-6R) [92] have also been identified as risk variants for AAA, pointing to possible impairment of macrophage function with respect to LDL handling [93] and ‘alternative’ phenotype switching [94]. As an abundant source of MMP-9, impaired macrophage function could prove vital in determining levels of MMP-9 release and therefore aneurysm growth. Unfortunately, GWAS findings have only identified a weak link between a variant in a region (20q13.12) which maps closely to the MMP-9 gene. However, an alternative hypothesis has been put forward based on the discovered association between the genetic variant at 20q13.12 and the *PLTP* gene nearby, which plays a vital role in high density lipoprotein metabolism (HDL) [95].

It is important to acknowledge the difficulty in determining genetic risk variants and their relevance for MMPs in AAA and AD, due to the redundant nature of various members of the MMP family. Particularly when shared, overlapping substrates exist between MMPs and allows for compensatory mechanisms to take over in the event of specific MMP loss [96]. As such, studies investigating the potential of AAA-relevant MMPs, such as MMP-2 and MMP-9, should focus instead on animal models and clinical trials rather than genetic association.

## 6. MMP2 and MMP9 in AAA and AD

**Figure 1:**
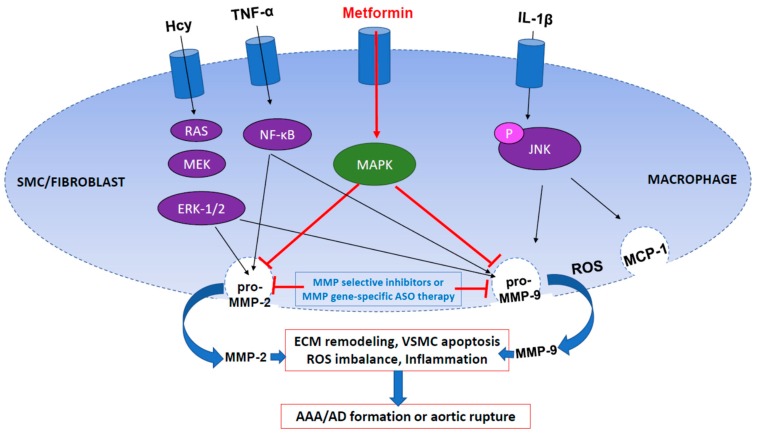
Signaling pathways involved in regulating MMP-2 and MMP-9 activity in VSMC/fibroblasts and macrophage, respectively. ASO: antisense oligonucleotide, Hcy: homocysteine, TNF-α: tumor necrosis alpha, IL-1β: interleukin 1 beta, MCP-1: monocyte chemoattractant protein 1, ROS: reactive oxygen species.

MMP2 and MMP9 are the two most critical players in AAA and AD development (Figure 1). Findings have shown that MMP-2 is predominantly derived from smooth muscles cells and fibroblasts, and to a lesser extent macrophages [87], whereas MMP-9 is predominantly derived from macrophages and to a lesser extent neutrophils [81,97]. Due to the multi-cellular origins of these two MMPs, attempts to block specific MMP function is arguably more favorable than attempting cellular blockade into the aneurysmal tissue. MMP9^-/-^ and MMP2^-/-^ mice were used to determine their effect on aneurysm formation. Compared to wild type mice, both groups of genetically modified mice showed no difference in aortic diameter size, 10 weeks after periadventitial application of CaCl_2_. Neither group showed reduced infiltration of macrophages, despite the absence of MMP2 to MMP9, pointing to a downstream role for these MMPs following their release into the aorta. Interestingly, infusion of wild type macrophages resulted in reconstitution of aneurysms in MMP9^-/-^ mice, but not MMP2^-/-^, highlighting MMP-9 specific release from macrophages, as expected [98].

The authors of this paper suggested that concerted activation of TGF –β may be one of the ways in which MMP-2 and MMP-9 work together. Although activation of the TGF –β signaling pathway is generally understood to offer protection against AAA development [99], with important roles in enhancing Type I and III collagen production [100], and increasing expression of protease inhibitors, plasminogen activator inhibitor-1 (PAI-1) [101] and TIMP-1 [102].

On the other hand, the MAPK (mitogen-activated protein kinase)/ERK pathway has been shown to play a clearly defined role in AAA formation. As stated previously, one member of the Mitogen-activated protein kinase (MAPK) pathway, JNK, has already been shown to be important in AAA formation by regulating MMP-9 activity [103]. Expression of another member, ERK-1/-2, was found to be upregulated in human aortic tissues isolated from AAA patients compared to controls. Moreover, knock down of ERK in mice (ERK-1^-/-^) prevented AAA formation by elastase perfusion, resulting in decreased activation of MMP-2 and MMP-9 [104], highlighting ERK as an upstream regulator of MMP-2 and MMP-9. Importantly, oxidative stress has been shown to play a role in triggering homocysteine-induced ERK1 activation leading to MMP-9 release from microvascular endothelial cells [105]. Further in vitro studies showed that pharmacological inhibition of ERK1/2 and p38 pathway, could attenuate MCP-1 mediated MMP9 release from human aortic smooth muscle cells [106]. One pathway has been investigated for its protective effect in AAA through MMP-2 and MMP-9 modulation. Activation of the AMPK signaling pathway was found to alleviate MMP-2 and MMP-9 expression as well as reduce expression of pro-inflammatory cytokines (TNF- α, IL-6, MCP-1, IL-1β) in AAA mice. Decreased phosphorylated AMPKα levels, typically observed in AAA patients, could be restored in mice infused with Angiotensin-II, using aminoimidazole-4-carboxamide-1-β-d-ribofuranoside (AICAR), a specific activator of the AMPK pathway, or metformin, a drug currently used in the treatment of Type II diabetes [107]. In light of this, metformin has now been proposed for clinical trial as a treatment for AAA (ClinicalTrials.gov Identifier: NCT03507413), and may also provide a helpful clue by explaining the reduced prevalence of AAA observed in patients with diabetes mellitus [108,109].

Importantly, different animal models of AAA have identified contrasting roles for MMPs including MMP-2. Shen et al. 2015 found that although MMP-2 was vital for promoting constructive remodeling of the thoracic aorta through enhanced ECM synthesis following Angiotensin-II infusion in mice, it enhances ECM degradation in the CaCl_2_ model [110]. These findings are further compounded by the fact that the role of MMP-2 in aneurysm development is subject to both regional and temporal circumstance. For instance in ascending thoracic aortic aneurysms (TAAs), a role for MMP2 has been widely established, however, its role in descending TAA is less clear [111]. Raised levels of MMP-2 within 72 h of aneurysm formation, followed by a rapid return to baseline levels would suggest a role for MMP-2 in early thoracic aneurysm formation only [112]. Moreover, the cellular source of MMP-2 and MMP-9 in aneurysmal development differs between the thoracic and abdominal aorta [113,114]. Thus, it is important to recognize these intrinsic differences when designing suitable therapeutic targets.

## 7. Other MMPs or TIMPS in AAA and AD

It is clear from the literature that multiple signaling pathways converge to regulate MMP-2 and MMP-9 activity within their respective cells (Figure 1). It is worth noting, however, that although much effort has been devoted to understanding their roles in AAA and AD pathology, many other MMPs are upregulated and enhance pathological processes under these conditions (Table 1). For instance, MMP-8 in conjunction with cathepsins K, L and S were found to be significantly upregulated in tissues sections isolated from asymptomatic and ruptured aneurysms. Importantly, these collagenases were identified as the key culprits of aneurysmal growth and rupture in which collagen turnover plays a huge part [115]. As such, pharmacological inhibition of lysosomal cathepsin proteases should also be considered for future pharmacological inhibition of AAA and AD [116]. Similarly, TIMPs play a key role in modulating MMP expression, as well as regulating leukocyte and VSMC behavior in AAA pathologies. However, conflicting findings add to the confusion arising from a complex and dynamic network of proteases [117,118,119,120]. Therefore, future studies are required, using comparable animal models, cell types, and experimental conditions, to determine the true role for each MMP and TIMP protein involved AAA and AD pathology.

## 8. Potential Applications of MMP Inhibition

As it stands, most pharmacological means of targeting MMP activity in AAA have consisted of targeting global MMP expression rather than through MMP-specific attenuation. Doxycycline has long been considered an effective non-selective MMP inhibitor by reducing gene expression [143], resulting in a reduced incidence of AAA in mice [144] and aortic growth in humans [145]. Interestingly salvianolic acid A has been shown to achieve similar levels of improved vascular integrity and decreased aortic diameter in ApoE^-/-^ mice infused with angiotensin II, however with reduced hepatoxocity levels compared to doxycycline treatment [146]. Moreover, the 3-hydroxy-3-methylglutaryl coenzyme A reductase inhibitor, cerivastatin, has been shown to suppress MMP-9 production in the aortic wall in humans [147] and prevent aneurysm formation in an elastase-induced AAA model in rats [148]. Conversely, the calcium channel blocker, amlodipine, has been shown to enhance MMP-9 activity and elastin degradation in porcine aortic segments [149]. Despite this, amlodipine was found to halt aortic dilatation in an angiotensin II-induced model of aortic aneurysm formation, however the absence of any MMP activity measurements in this study may suggest an alternative mechanism, such as blood pressure lowering, was responsible for reduced dilatation [150]. Another study found that in combination with the 3-hydroxy-3-methylglutaryl coenzyme A reductase inhibitor, atorvastatin and amlodipine could reduce MMP-2 activity in an angiotensin II infusion animal model of AAA [151]. Finally, imidapril, an angiotensin-converting enzyme inhibitor, has also been shown to attenuate aortic expansion in an elastase-induced model of AAA both in wildtype mice and further in angiotensin II type I (AT1) receptor knock out mice. Imidapril was found to achieve this without affecting blood pressure. As evidenced above, a number of pre-existing drugs have been tested for their efficacy at targeting aneurysm formation, with varied success. Future work should focus on MMP-specific attenuation, for instance, using synthesized molecules such as small molecular weight MMP inhibitors (MMPis) which work through chelation of the MMP ZN^2+^ ion active site and may offer potential therapeutic tools for targeting specific MMP activity during aneurysm formation [152].

## 9. Conclusions and Future Perspectives

First described in 1962, MMPs have been a huge source of interest as new functions and catalytic mechanisms become assigned to them in various physiological and pathological conditions [153]. Due to their abundance and multi-faceted nature, they offer a large scope for modulating key pathological processes in AAA and AD, particularly with regards to ECM turnover. As discussed previously, the cellular source of MMP-2 and MMP-9 in aneurysmal development differs at different locations [113,114], suggesting a distinct or in some occasions even an opposite contributions of different cellular sources of MMPs to AAA or AD. Unfortunately, MMP gene global knockout mice were used to examine the potential contribution of individual MMP to AAA and/or AD in the majority of studies. Although some researchers attempted to use alternative strategies (e.g., bone marrow transplantation [98,124,144] and cellular depletion [46]) to address above issue, cell lineage conditional MMP gene knockout mice would be more desirable tool for us to confirm the cell-specific roles of carious MMPs in AAA or AD. Moreover, under normal conditions, MMP-mediated ECM turnover allows cell migration within healthy tissues, vital for maintaining structural integrity of the aorta. As such, effects observed through global interference of MMPs in murine models of AAA provide a somewhat unrealistic portrayal of outcomes to be expected in humans, since localized MMP inhibition is more desirable. Hence, future investigations using drug-eluting grafts or flow diverting stents following EVAR procedures may present one localized method of preventing aneurysmal growth and aortic rupture following AD [154]. However, this still does not offer a substantial solution to preventing enlargement of small aneurysms, reducing the need for patients to undergo surgery in the first place.

As such, alternative methods have been investigated including delivery of rapamycin via nanoparticles in an elastase infusion model of AAA. Accumulation of rapamycin was found to successfully target aortic aneurysms and reduce the expression of MMPs and inflammatory cytokines [155]. Similarly, delivery of nanoparticles loaded with batimastat (a hydroxamate-based MMP inhibitor) was found to be more effective at reducing MMP activity, elastin degradation and aortic wall expansion in a CaCl_2_ murine model of AAA compared with batimastat administration alone. Nanoparticle-based MMP inhibition studies have demonstrated numerous benefits in the treatment of AAA including improved drug delivery, reduced off-target effects and in some cases a reversal of disease pathology [156,157].

Future studies using a similar delivery system but targeting specific MMP gene expression through oligonucleotide-based therapy may prove to be an ideal method of understanding localized, MMP-specific responses to the highly inflammatory and hypoxic environment in the aorta during AAA and AD [158]. It is clear from the literature that protease contributions to AAA and AD pathology are immense and targeted inhibition could significantly improve the prognosis for aneurysm patients. However, significantly more work must be carried out to improve our understanding of specific MMP and TIMP involvement, particularly with regards to their signaling pathways and temporal influence on disease progression. Once their contributions have been clearly established, appropriate delivery methods for modulating their activity will need to be considered as well.

## Figures and Tables

**Table 1:** Other MMPs or TIMPS in AAA and AD.

	Role within AAA, AD or Rupture	Substrate	Stimulus	Known Signaling Pathways	Ref.
MMP-1	Associated with increased rates of aneurysmal rupture and reduced survival, and aortic dissection	Collagen triple helix	TNF-α IL-1	(MAPK family) JNK, ERK and p38 kinase-induced activation of MMP-1	[121,122,123]
MMP-3	Activity promotes AAA	Elastin	Netrin-1	Netrin-1 binds neogenin-1 receptors on VSMCs to activate MMP3	[124]
MMP-7	Increased expression in AAA	N-cadherin		PI-3 kinase/Akt VSMC apoptosis and proliferation	[125]
MMP-8	Elevated expression in growing and rupture AAA, released by neutrophils	Collagen triple helix Cystatin C	Ox-LDL IL-1		[115,126]
MMP-12	Promotes AAA growth by regulating leukocyte recruitment Promotes AD formation	Elastin CXC-chemokine ligand 2 and 3 IFNγ	IL-3	MMP-12 cleaves N-cadherin, triggering ß-catenin signalling and VSMC proliferation	[127,128,129,130,131]
MMP-13	Elevated expression in AAA sections and thoracic aortic dissection tissues, predominantly localised to VSMCs	Collagen triple helix	TNF-α IL-1	(MAPK family) JNK, ERK and p38 kinase-induced activation of MMP-13	[132,133]
MMP-14 (MT1-MMP)	Modest increase in tissues of ruptured AAA	Collagen triple helix			[134]
MMP-17 (MT4-MMP)	Inhibits AAA formation	Osteopontin in VSMCs		c-Jun N-terminal kinase signalling, VSMC maturation	[135]
MMP-19	Expression is associated with aneurysms				[134]
TIMP-1	Increased levels in AAA Overexpression leads to ablation of AAA within experimental rodent model Deletion enhances aneurysm formation	MMP-1, MMP-9 and MMP-3	TNF-α	Inhibits MMP-1, MMP-9 and MMP-3	[97,136,137,138,139]
TIMP-2	TIMP-2 promotes aortic growth through activation of MMP-2 in murine model of AAA Reduced expression of TIMP-2 in late stage cerebral aneurysm formation	MMP-2		Regulates MMP-2	[117,118]
TIMP-3	Increased expression in response to MMP over activity, with heightened expression in human AAA end stage tissues Loss of TIMP-3 leads to worsening of the AAA pathology Reduces VSMC proliferation and migration	MMP-2, MMP-9, TNF-α	TGF-ß		[140,141,142]
